# Supplementary material for: Spinal cord injury enhances lung inflammation and exacerbates immune response following exposure to LPS
Source: Front Immunol. 2025 Jan 15;15:1483402. doi: 10.3389/fimmu.2024.1483402 (PMC11774706; doi:10.3389/fimmu.2024.1483402)
Supplement: Supplementary file 1 [file DataSheet1.docx]

**Title: Spinal Cord Injury Enhances Lung Inflammation and Exacerbates Immune Response Following Exposure to LPS**

**Running title: Spinal cord injury enhances lung inflammation**

**SUPPLEMENTAL FIGURE 1. Spinal cord injury displacement and impact force.**

Sixteen C57BL/6N mice underwent T9 spinal cord injury (SCI) surgery. (A) SCI displacement and (B) SCI severity were recorded (n=8 per group). Ten days post-SCI, mice were subjected to either acute lung injury (ALI) or no ALI. Data were expressed as mean ± SEM.
